# Letter from J. O. Martin

**Published:** 1852-07

**Authors:** J. O. Martin


### CORRESPONDENCE.

Franklin, Ind., June 22d, 1852.

Dr. Taylor—Dear Sir:—I wish to lay before you the particulars of a case in which Chloroform was given mixed with an equal quantity of ether. About the 18th or 19th of May, Mr. Clements a gentleman of this place, (Franklin,) wished to have some three or four teeth extracted, and for this purpose called on Dr. Haynes who was at the time delivering a course of lectures on Phrenology, in this place; in addition to this, he advertised to extract and fill teeth on the most modern and improved plan.

Mr. Clemens says, that the mixture filled a pint bottle, and that the Dr. allowed him to inhale the Chloroform until he was so far under the influence that the Dr. could not get the forceps in his mouth; he therefore, had to wait until he got over the influence a little and then would give him a little more, until Mr. Clemens says the pint was disposed of in this way before the teeth were all extracted. The Chloroform and ether was purchased at the Drug Store of Mr. Moore, of this place, and was mixed together by Mr. Moore, at the direction of Dr. Haynes. Mr. Moore says he has forgotten the quantity sold. Mr. Clemens says that soon after the above operation his memory became very much impaired; so much so, that when he would start to get something about the house, he would entirely forget what it was before crossing the room. And on the 7th or 8th day after taking the mixture, he experienced a very singular sensation about the eyes, the muscles became stiff and contracted so that it was with difficulty he could close his eyes. About the ninth day after taking the mixture, he discovered that his head was full of dandruff, as he supposed, but on examination it was found that the skin covering the head was all peeling off in large white scales. About this time he experienced a very disagreeable sensation in all the muscles of the body and extremities if he would attempt to sit down. He says it appeared as though there was a thousand sharp needles running in him. He further states, that when he would take a drink of cold water it would produce a shock similar to a Galvanic Battery; in one or two days after this, and about the 10th or 11th day after the operation, the skin on the body and extremeties commenced peeling off in large white scales which produced an itching sensation, and where he happened to rub the skin it became very much inflamed and quite sore. It has now been little more than one month since Mr. Clemens had his teeth extracted, and those disagreeable feelings are passing off and he is attending to his business as usual, but he has been skinned certain.

Being a Dentist myself, and having been in the habit of giving Chloroform occasionally, and not having met with anything similar to this in my practice, I concluded to lay the matter before you, and if you think proper you can make such comments upon it as you may think proper.

Yours respectfully,
J. O. MARTIN.

---

We give the above, regarding the singular symptoms, as the result of the large quantity administered. We have, however, never met with such symptoms after the administration of either ether or Chloroform, separately or combined. But we have never given it in such doses and think we should desist before we had given much over an *ounce*. We could account for the impaired memory, more satisfactorily than for the after skinning which the patient received.—Ed.

